# Supplementary material for: The impact of curcumin derived polyphenols on the structure and flexibility COVID-19 main protease binding pocket: a molecular dynamics simulation study
Source: PeerJ. 2021 Jul 19;9:e11590. doi: 10.7717/peerj.11590 (PMC8297469; doi:10.7717/peerj.11590)
Supplement: Supplemental Information 1 [file peerj-09-11590-s001.pdf]

The impact of natural polyphenol on the structure and flexibility COVID-19 main protease binding pocket: a molecular dynamics simulation study

Aweke Mulu Belachew[1*], Mulugeta Gajaa Ufgaa[2] and Jerusalem Fekadu W/ Mariam3

[1]Deprtment of Industrial Chemistry, college of Applied Science, Addis Ababa Science and Technology University, Addis Ababa, Ethiopia

[2]Department of Physics and Statistics, College of Natural and Social science, Addis Ababa Science and Technology University, Addis Ababa, Ethiopia

[3] Department of Eco biology, college of Applied Science, Addis Ababa Science and Technology University, Addis Ababa, , Ethiopia

Corresponding Author:

Aweke Mulu Belachew1*

Addis Ababa, Ethiopia, 16417, Ethiopia

Email address: aweke.mulul@aastu.edu.et / awekemulu@gmail.com

Table 1. Details of the selected hits. Open BABEL structure and docking energies obtained after virtual screening and refinement by molecular docking are shown.

Compound	*Structure*	Autodock (Kcal.mol^{-1})	Autodock Vina (Kcal.mol^{-1})
Diferuloylmethane	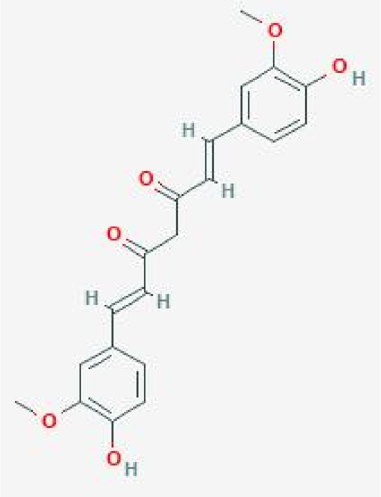	-7.98	−202.476 kcal/mol

demethoxycurcumin	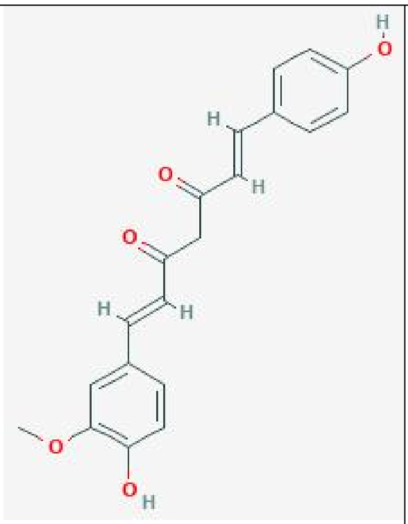	-8.17	103.901 Kcal/mol
Bisdemethoxycurcumin	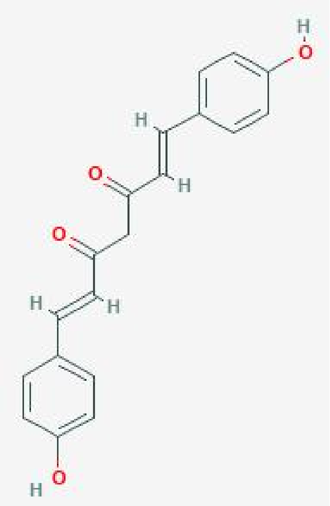	-8.30	−151.545 Kcal/mol

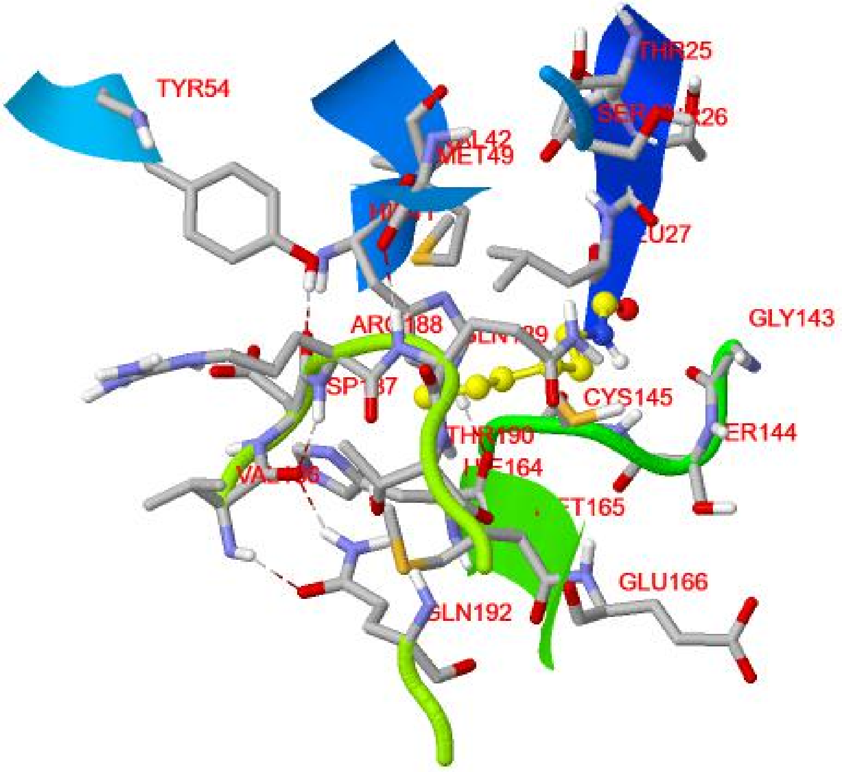

Figure 1. The potential binding position for the Carmfur-Mpro complex of binding modes in the active site (residues are shown in sticks). The red dotted line represents intermolecular hydrogen bond interactions.

Table 2. Table showing the Van der Waal, electrostatic, polar salvation and binding energy in kJ mol^{-1} for Mpro and its Ligands in complex with Carmfur.

Ligand - enzyme complex	Electrostatic energy (kJ/mol)	Van der Waal energy (kJ/mol)	Polar solvation energy (kJ/mol)	Binding free energy (kJ/mol)
Enzyme - bisdemethoxycurcumin complex	−282.1 ± 1.1	−125.1 ± 1.4	264.8 ± 2.4	−169.7 ± 2.5
Enzyme-demethoxycurcumin complex	-118.4 ± 3.7	-108.1 ± 2.2	162.8 ± 5.9	-89.0 ± 45.3
Enzyme-diferuloylmethane	-339.1 ± 15.6	-364.2 ± 18	492.6 ± 15.2	-39.0 ± 1.5

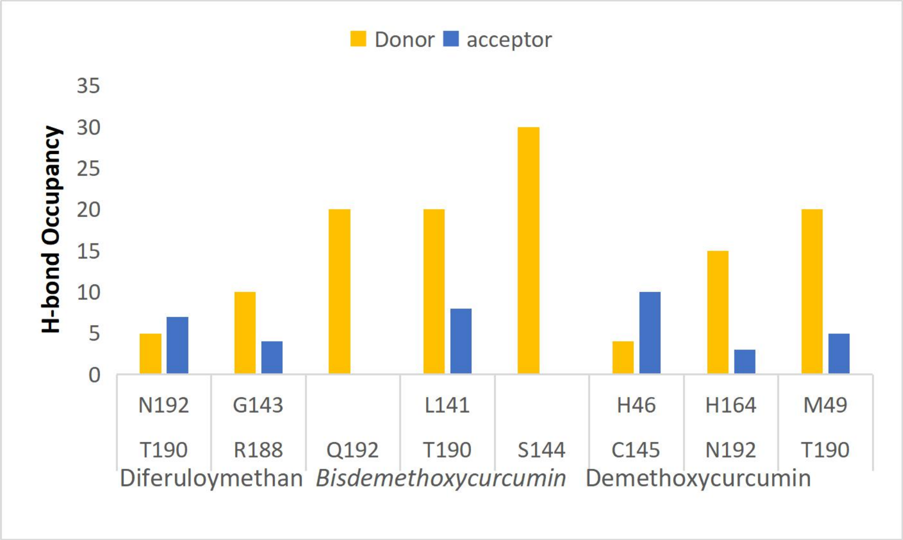

Figure 2. Hydrogen bond occupancy analysis for Bisdemethoxycurcumin, Demethoxycurcumin and diferuloylmethane.
