# Supplementary material for: The impact of curcumin derived polyphenols on the structure and flexibility COVID-19 main protease binding pocket: a molecular dynamics simulation study
Source: PeerJ. 2021 Jul 19;9:e11590. doi: 10.7717/peerj.11590 (PMC8297469; doi:10.7717/peerj.11590)
Supplement: Supplemental Information 12 [file peerj-09-11590-s012.docx]

Results

Table 1 shows molecular docking results to determine the SARS CoV-2 main protease (Mpro) binding pocket affinity towards selected curcumin sp derived polyphenols at 298.15 K

| Protein-ligand complexes | Binding Energy (kcal/mol) | Inhibition Constant (Ki) (uM) | RMSD ^b^ (A^0^) | Residues involved in interaction | No of Hydrogen Bonds |
| --- | --- | --- | --- | --- | --- |
| Diferuloylmethane -COVID-19 main protease | -12.25 | 156.42 | 70.645 | Asn142 and Gln192 | 2 |
| Demethoxycurcumin-COVID-19 main protease | -12.17 | 281.22 | 57.842 | Leu272, Thr199,Lys137 | 3 |
| Bisdemethoxycurcumin-COVID-19 main protease | -12.32 | 98.83 | 65.030 | Phe294, Gln110 and Glu240 | 3 |


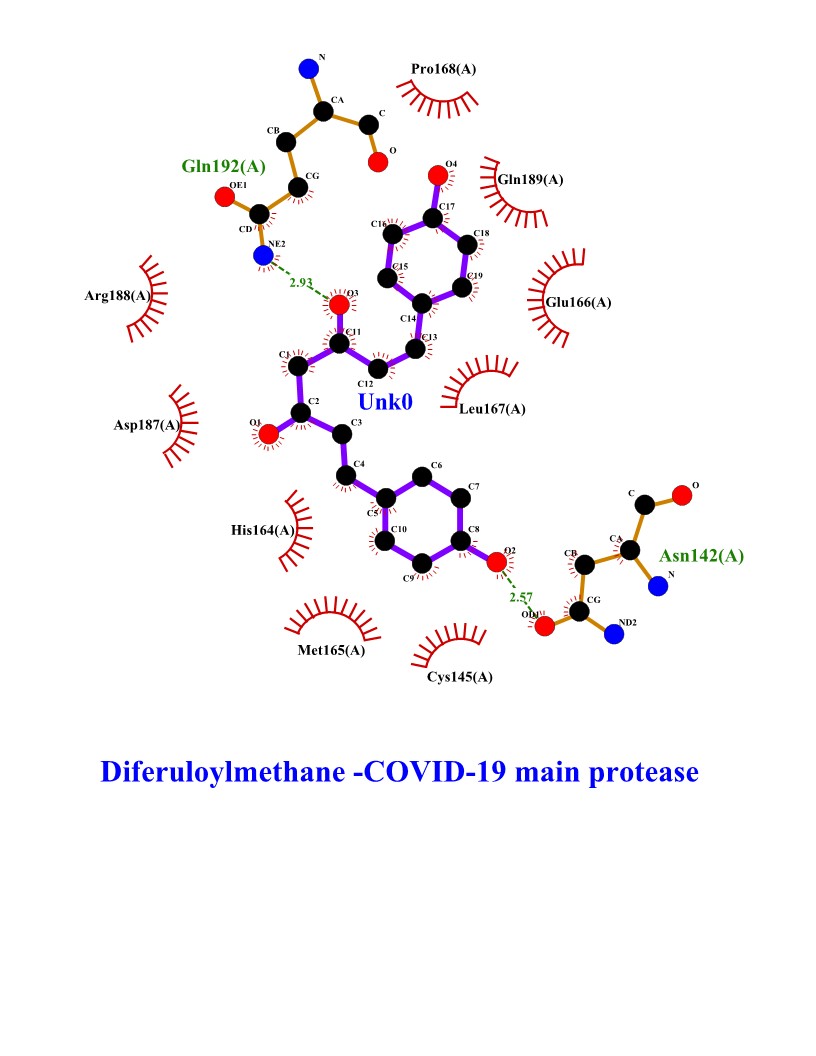


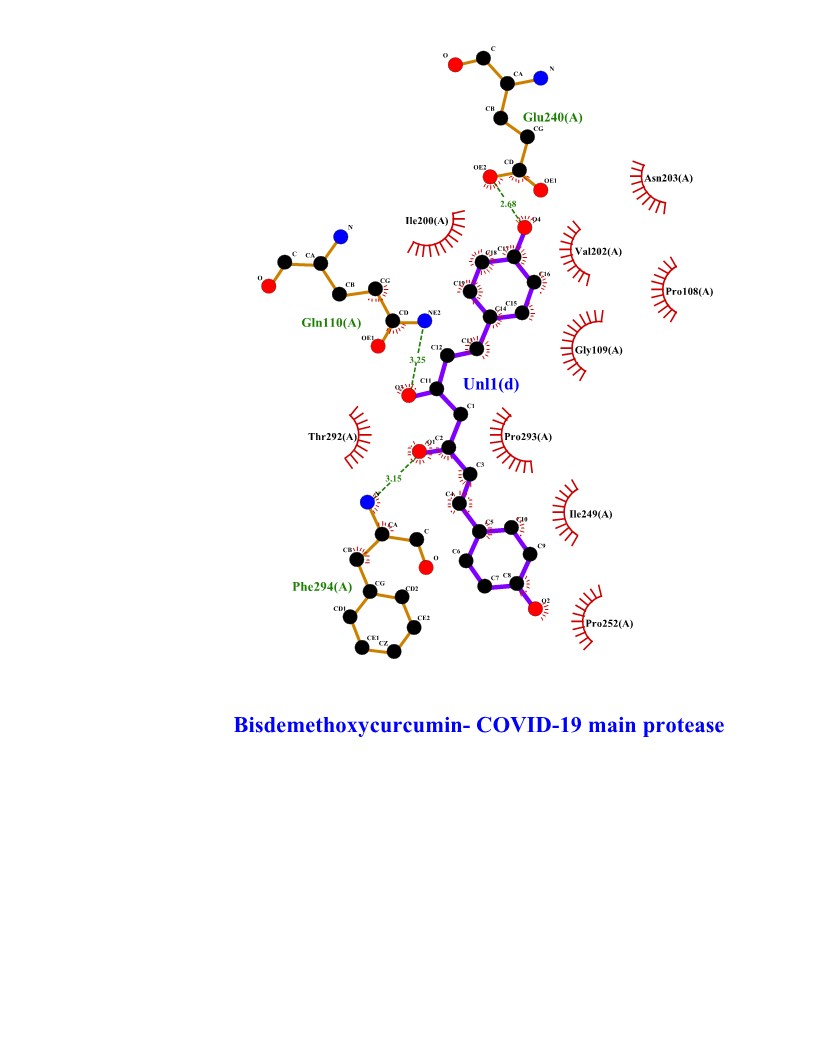

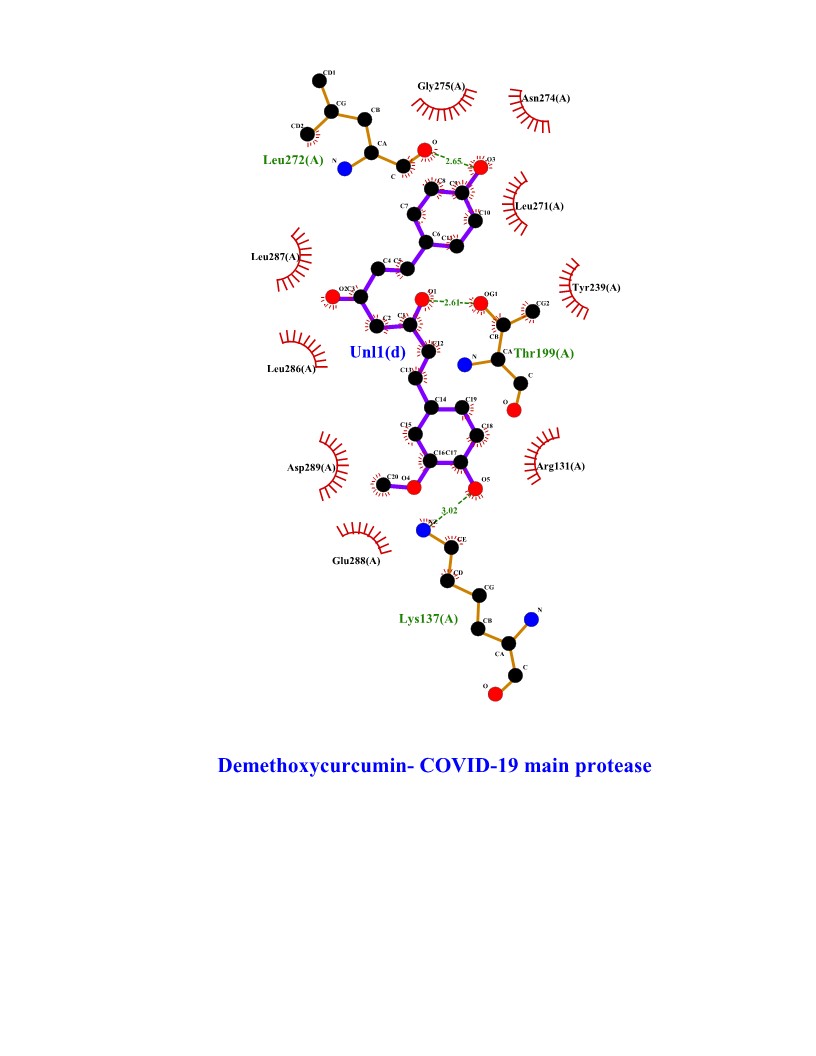


Figure 1 Interactions of selected polyphenols and Main protease binding pocket residues. For all polyphenols, carbon atoms are shown in black, oxygens in red, and nitrogens in blue. Bonds in the Diferuloylmethane, Demethoxycurcumin, and Bisdemethoxycurcumin are shown in purple, and bonds in binding pocket residues are in brown. Hydrogen bonds are shown (with their lengths) as green dashed lines. Residues making hydrophobic interactions with the Diferuloylmethane, Demethoxycurcumin, and Bisdemethoxycurcumin are shown as red arcs with radiating lines. Diferuloylmethane, Demethoxycurcumin, and Bisdemethoxycurcumin atoms involved in these hydrophobic interactions are shown with radiating red lines


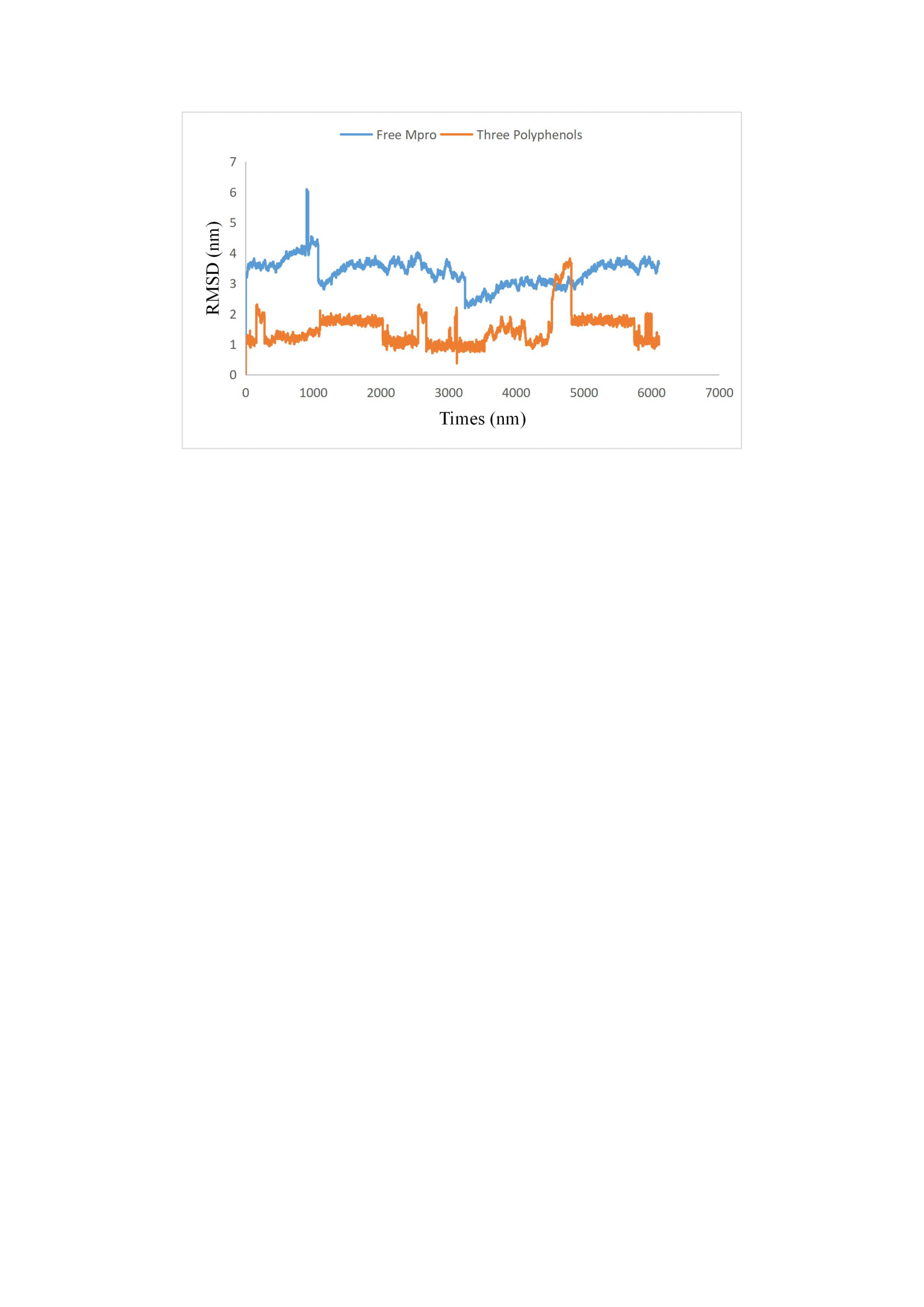


Figure 2 Plots of Root-mean-square deviations of free SARS CoV-2 main protease (Mpro) (Blue) and the complex of SARS CoV-2 main protease (7BUY) (Red) with three polyphenols along the MD simulation time.


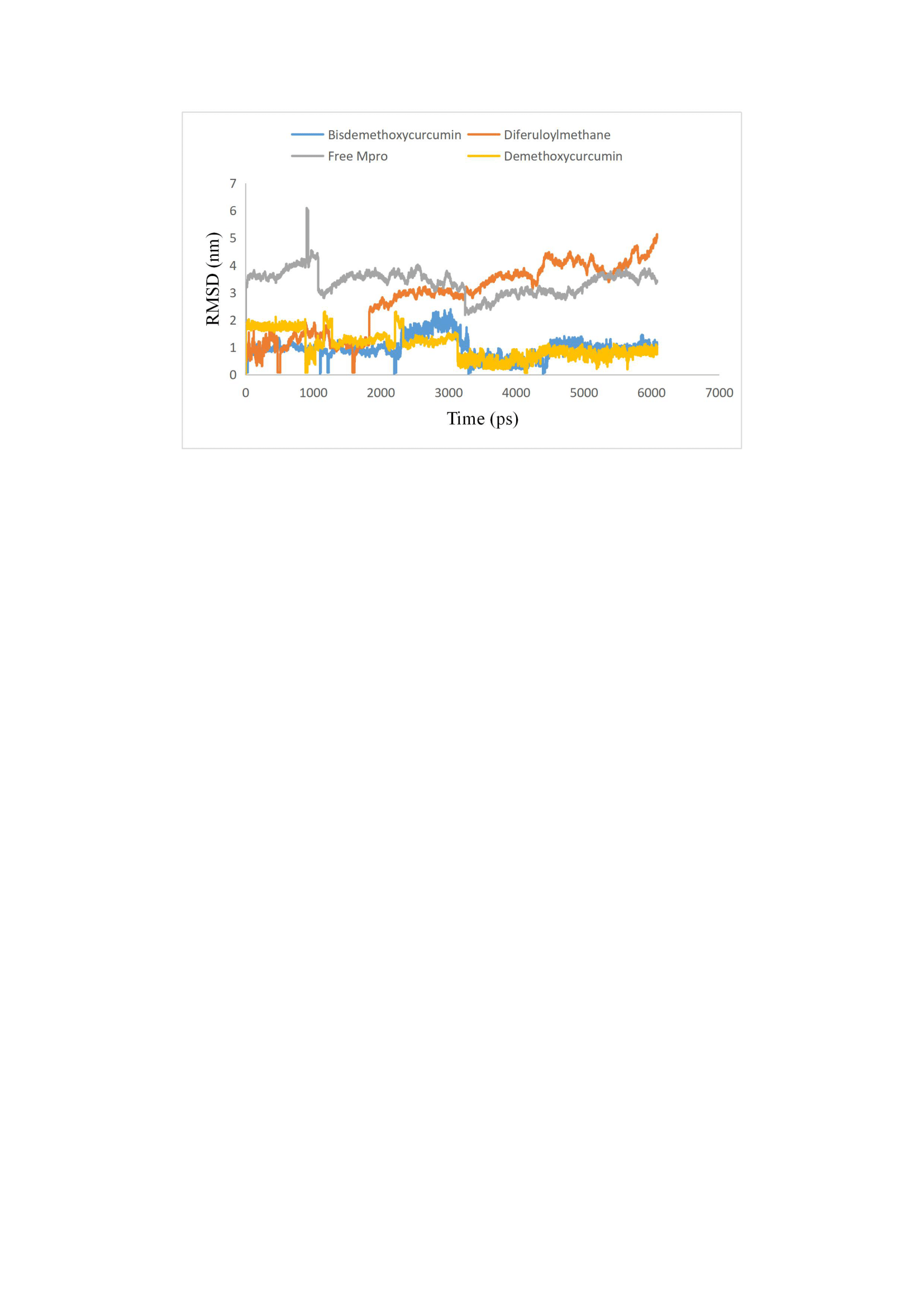


Figure 3 Plots of Root-mean-square deviations of free main CoV-2 protease (Mpro) (Gray) and the complex of Mpro with Bisdemethoxycurcumin (Yellow), Demethoxycurcumin (Blue) and Diferuloylmethane (Red) along the MD simulation time for three individual polyphenols.


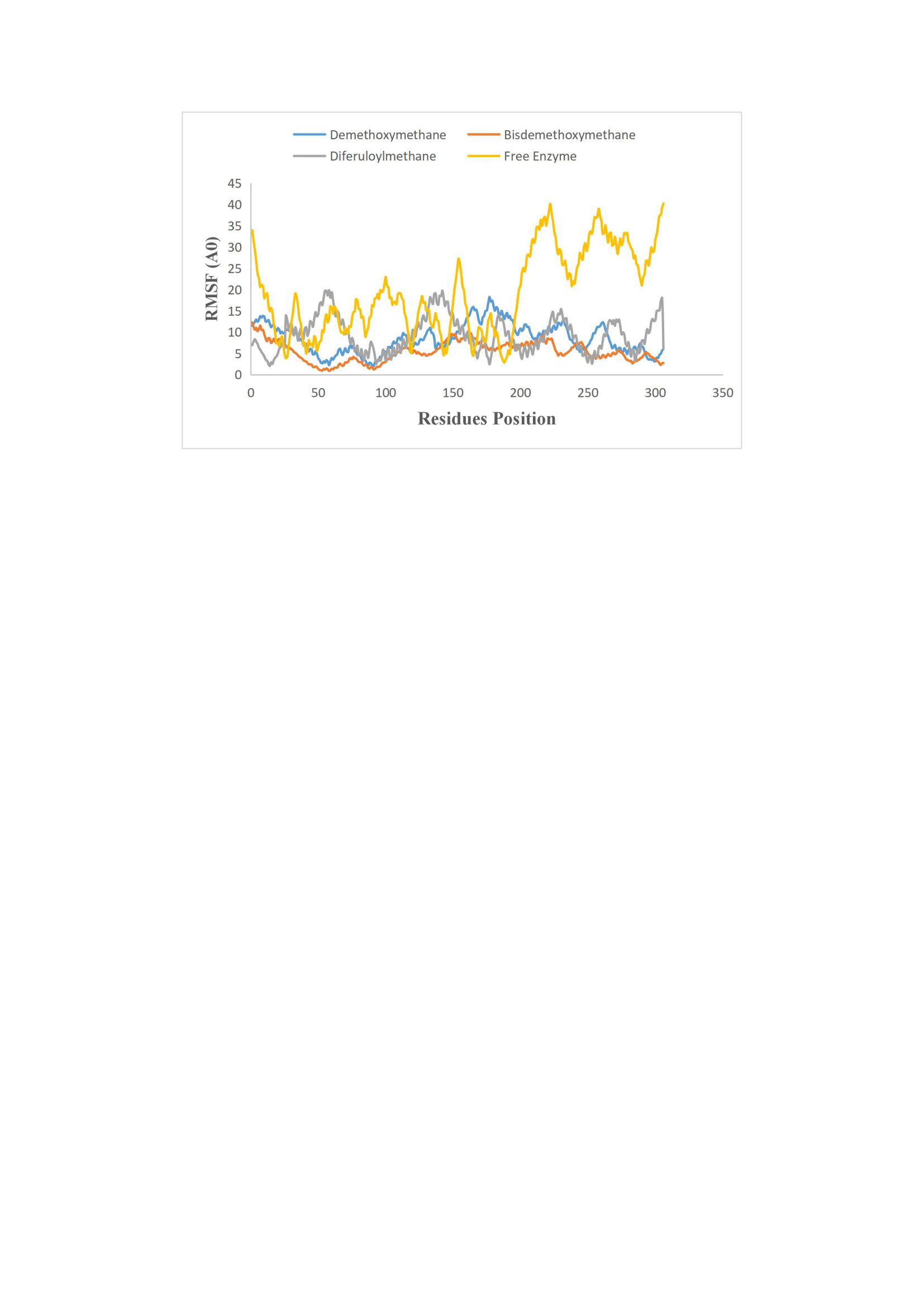


Figure 4 RMSF plot of free main CoV-2 protease (Mpro)(Yellow) and the complex of Mpro with Bisdemethoxycurcumin (Red), Demethoxycurcumin (Blue) and Diferuloylmethane (Gray) along the MD simulation time for three individual polyphenols.


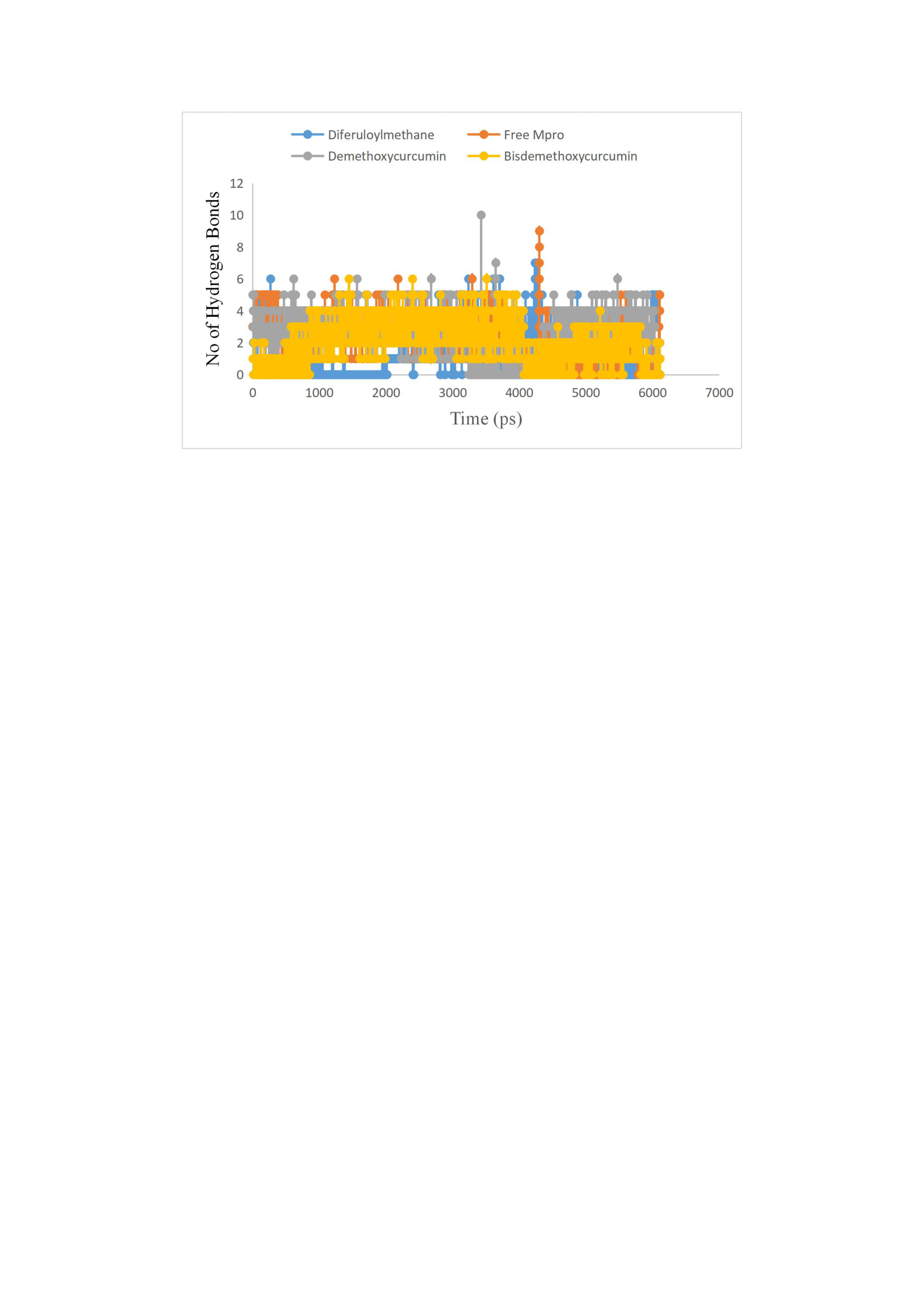


Figure 5 Number of hydrogen bond present in Bisdemethoxycurcumin-SARS-CoV-2 main protease (Yellow), Demethoxycurcumin-SARS-CoV-2 main protease (Gray), free SARS-CoV-2 main protease (Red) and diferuloylmethane-main protease (Blue).

Table 2 Inter-residue distances and corresponding standard deviations. In each residue pair, one belongs to the binding pocket loop region and the other belongs to surface part of the enzyme

| Residues | Free enzyme | Enzyme-Demethoxycurcumin complex | Enzyme-Bisdemethoxycurcumin complex |
| --- | --- | --- | --- |
| Arg188- Phe294 | 5.154 ± 1.531 | 5.937 ± 1.604 | 4.5 ± 1.10 |
| Val202- Gln110 | 9.419 ± 1.440 | 8.455 ± 1.090 | 9.25 ± 1.440 |
| Gly109- Gln240 | 10.111 ± 1.838 | 8.296 ± 1.459 | 11.31 ± 1.14 |
| Pro293- Gln192 | 8.570 ± 1.663 | 7.095 ± 1.022 | 5.13 ± 1.04 |
| Ile249- Asn142 | 9.170 ± 5.015 | 10.485 ± 1.560 | 6.71 ± 0.44 |
| Pro252- Lys137 | 7.11 ± 0.02 | 8.5 ± 1.4 | 5.63 ± 1.04 |
| Thr292- Thr199 | 5.109 ± 0.440 | 6.039 ± 0.940 | 4.110 ± 0.230 |
| Ile200- Leu272 | 7.213 ± 0.018 | 9.216 ± 0.218 | 6.121 ± 0.618 |

Table 3 residues forming contacts with diferuloylmethane polyphenol, demethoxycurcumin polyphenol and bisdemethoxycurcumin polyphenol in binary complexes.

| Residues | Enzyme-Demethoxycurcumin complex | Enzyme Bisdemethoxycurcumin complex | Enzyme-diferuloylmethane complex |
| --- | --- | --- | --- |
| Gln192 | 0.70 ± 0.01 | 0.85 ± 0.01 | 0.45 ± 0.02 |
| Leu167 | 0.90 ± 0.03 | 0.98 ± 0.004 | 0.60 ± 0.05 |
| Tyr239 | 0.60 ± 0..07 | 0.80 ± 0.01 | 0.30 ± 0.01 |
| Asp289 | 0.70 ± 0.19 | 0.90 ± 0.004 | 0.60 ± 0.1 |
| Arg188 | 0.75 ± 0.011 | 0.80 ± 0.05 | 0.73 ± 0.09 |
| Glu166 | 0.80 ± 0.04 | 0.80 ± 0.01 | 0.90 ± 0.01 |
| Glu288 | 1.00 ± 0.06 | 0.50 ± 0.01 | 0.60 ± 0.12 |
| His114 | 1.00 ± 0.00 | 1.00 ± 0.00 | 0.50 ± 0.03 |
| Gln189 | 0.70 ± 0.05 | 0.90 ± 0.00 | 1.00 ± 0.09 |
| Pro168 | 0.30 ± 0.01 | 1.00 ± 0.00 | 0.85 ± 0.05 |
| Gln189 | 0.45 ± 0.02 | 1.00 ± 0.00 | 0.40 ± 0.16 |
| Cys145 | 0.75 ± 0.09 | 0.90 ± 0.01 | 0.60 ± 0.06 |
| Met165 | 0.80 ± 0.1 | 0.85 ± 0.12 | 0.50. ± 0.12 |
| His 164 | 0.40 ± 0.00 | 0.70 ± 0.01 | 1.00 ± 0.04 |
| Asp187 | 0.90 ± 0.03 | 0.90 ± 0.03 | 0.90 ± 0.03 |
| Arg188 | 0.93 ± 0.12 | 0.89 ± 0.01 | 0.76 ± 0.23 |
