# Supplementary figures and images for: The impact of curcumin derived polyphenols on the structure and flexibility COVID-19 main protease binding pocket: a molecular dynamics simulation study
Source: PeerJ. 2021 Jul 19;9:e11590. doi: 10.7717/peerj.11590 (PMC8297469; doi:10.7717/peerj.11590)

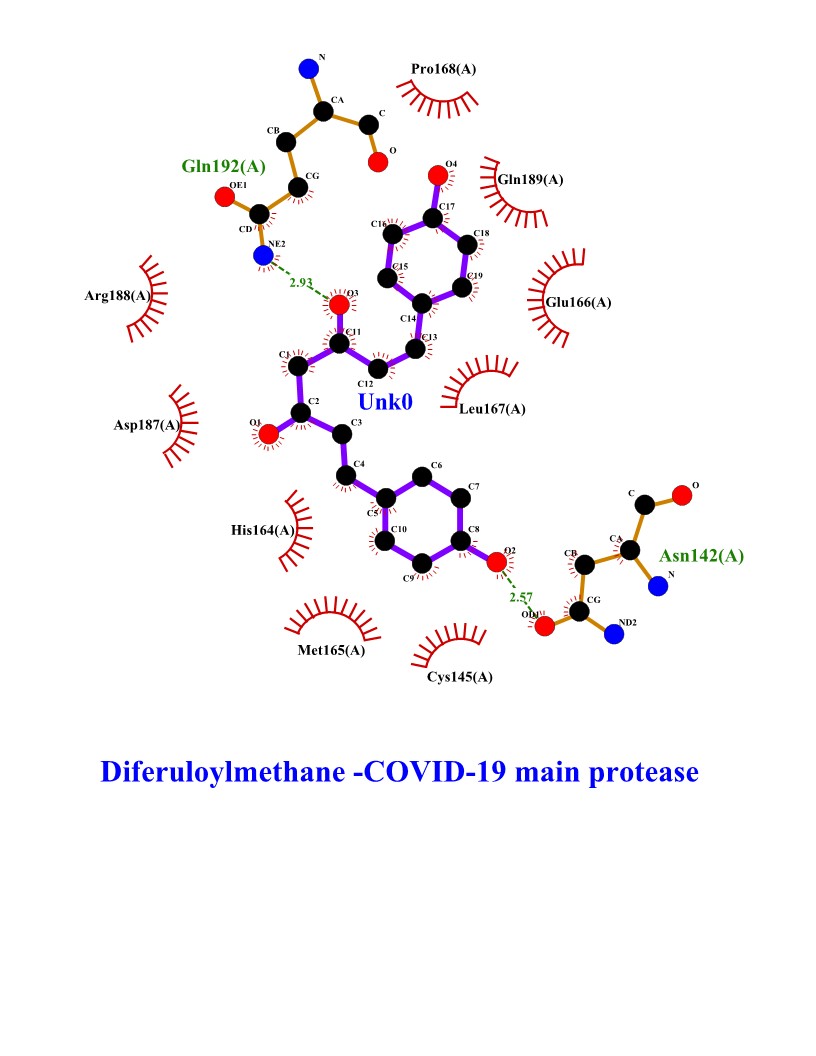


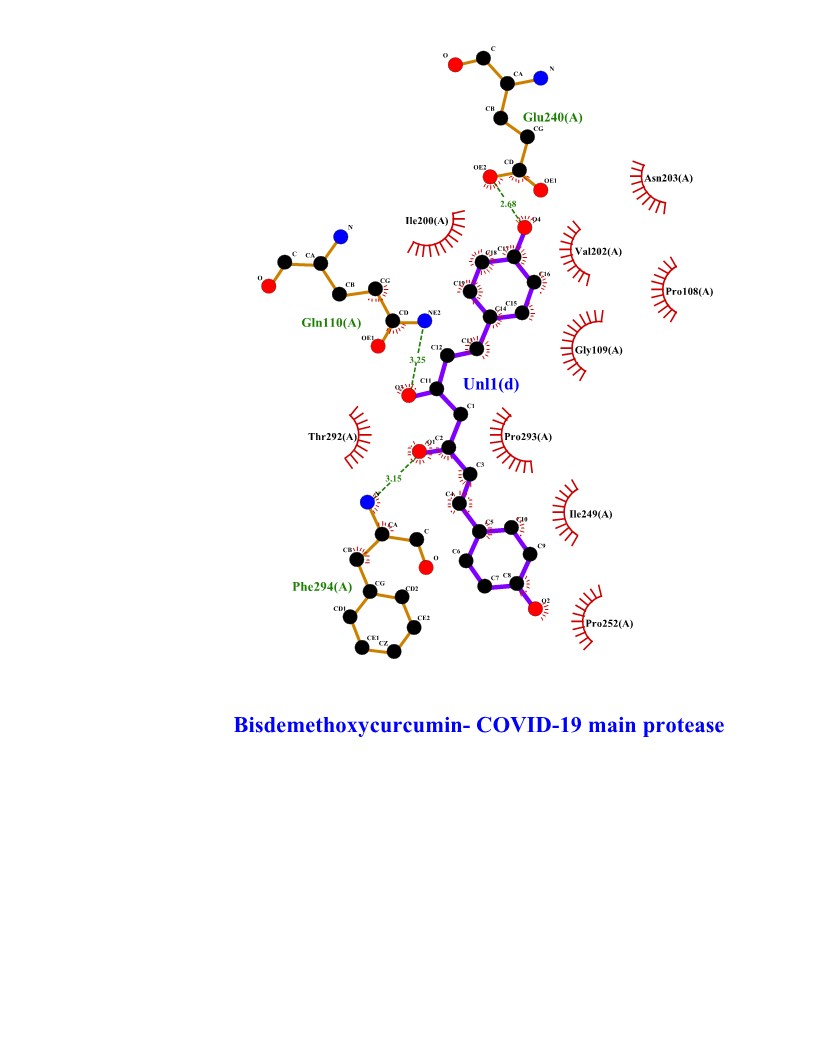

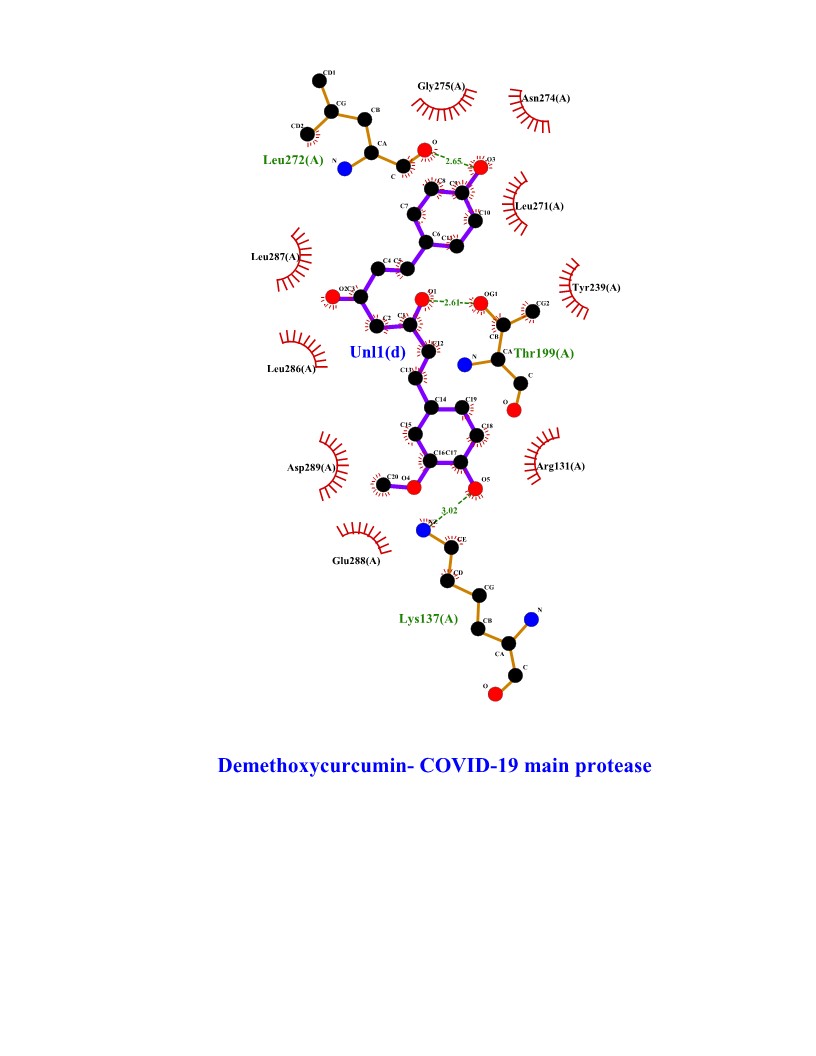

Supplement: Supplemental Information 13 [file peerj-09-11590-s013.docx]

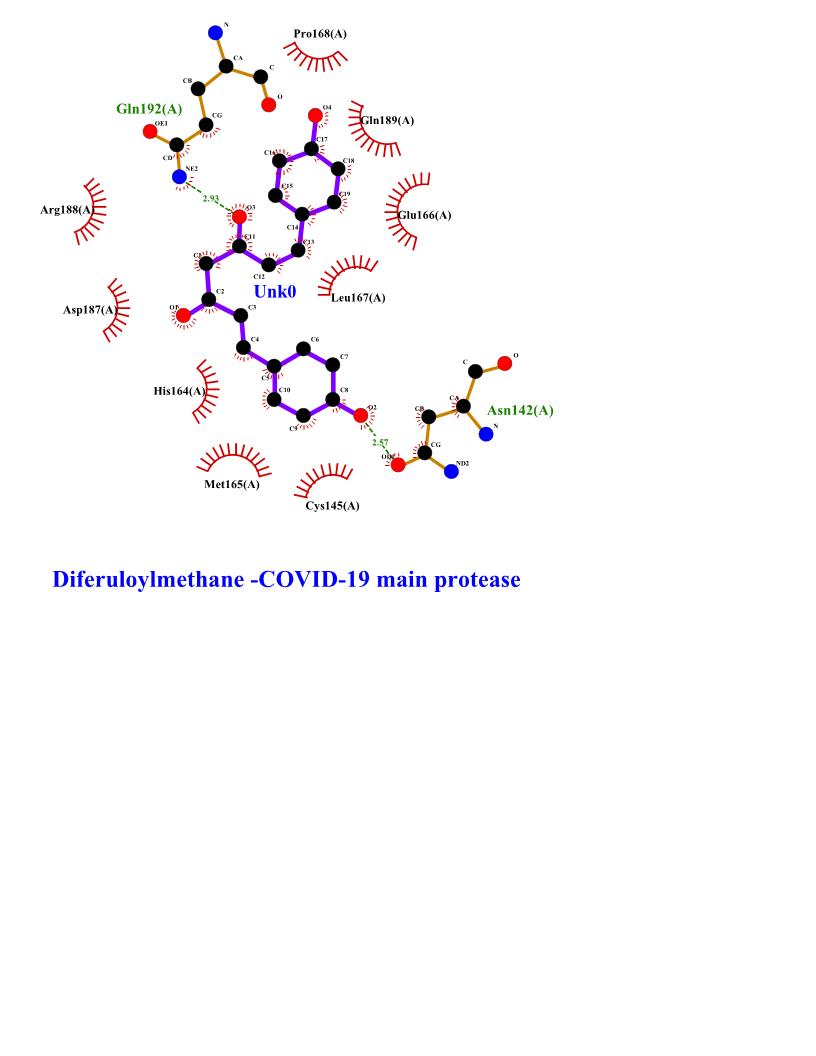

Supplement: Supplemental Information 14 [file peerj-09-11590-s014.png]

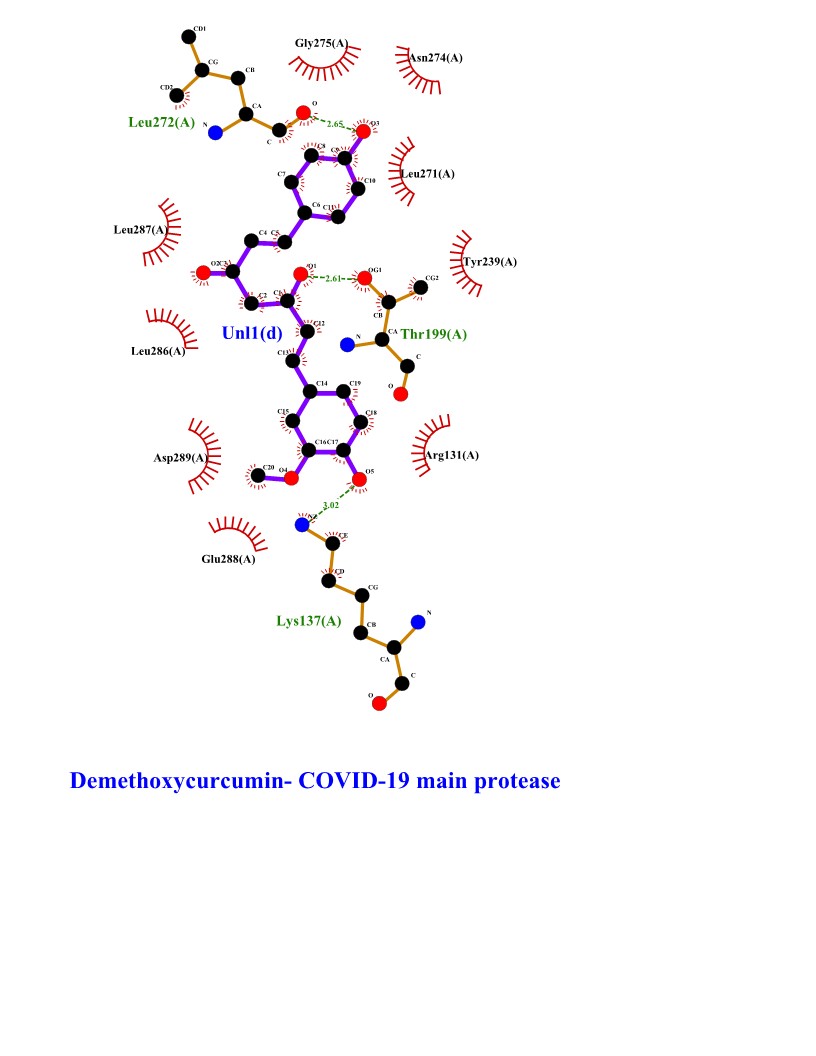

Supplement: Supplemental Information 15 [file peerj-09-11590-s015.jpg]

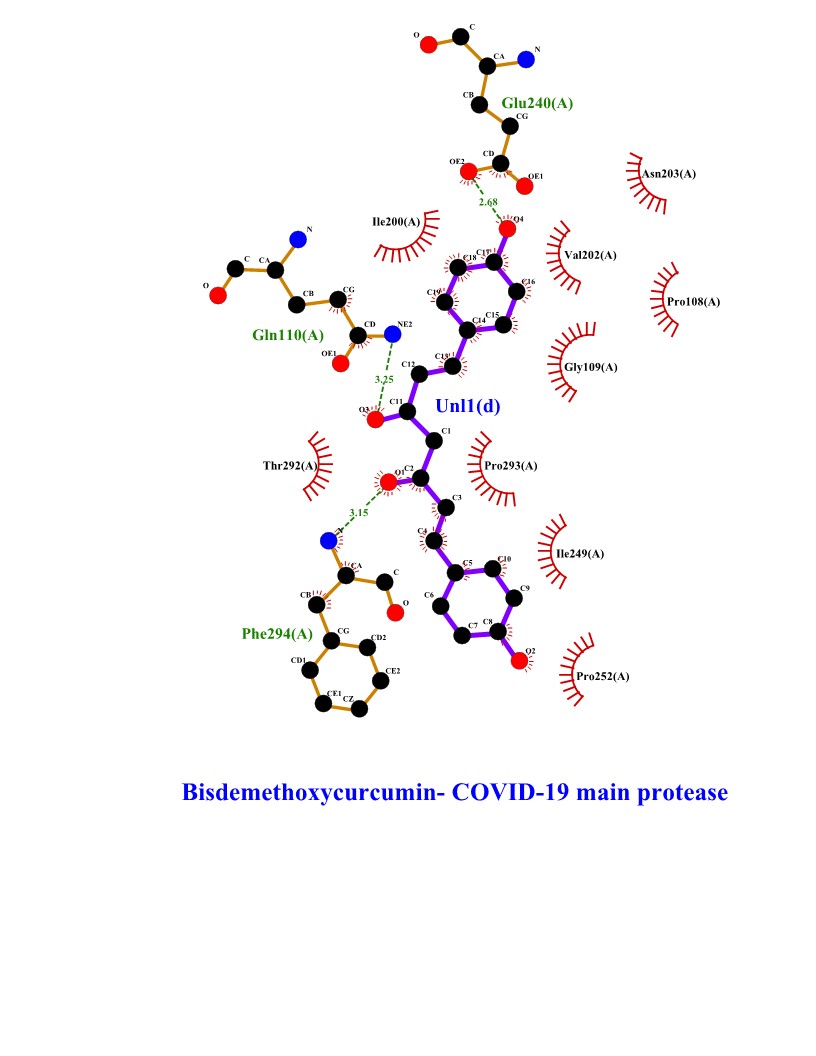

Supplement: Supplemental Information 16 [file peerj-09-11590-s016.jpg]
